# Hemmeter

**Published:** 1900-09

**Authors:** 


HEMMETER.—Diseases of the Stomach, their Special Pathology, Diagnosis, and Treatment, with Sections on Anatomy, Physiology, Chemical and Microscopical Examination of Stomach Contents, Dietetics, Surgery of the Stomach, etc. By John C. Hemmeter, M. D., Professor in the Medical Department of the University of Maryland, Baltimore. With many original illustrations, a number of which are in colors. Second edition, enlarged and revised. Octavo. 898 pages. Price, $6.00 net, cloth. P. Blakiston's Son & Co., 1012 Walnut Street, Philadelphia, Pa.

The subject of this book has within the last few years been greatly simplified and made practical. It may yet be some time before the average practitioner takes advantage of all that is now known about the diseases of the stomach, but there is much that is practically new and eminently useful that can be utilized in routine practice. This work is a most satisfactory treatise, and brings the subject up to date. It includes much original work and is particularly clear and practical. T. J. B.

